# Isolation and Rheological Characterization of Cellulose Nanofibrils (CNFs) from Coir Fibers in Comparison to Wood and Cotton

**DOI:** 10.3390/polym10030320

**Published:** 2018-03-14

**Authors:** Daran Yue, Xueren Qian

**Affiliations:** Key Laboratory of Bio-Based Materials Science and Technology of Ministry of Education, Northeast Forestry University, No. 26 Hexing Road, Xiangfang District, Harbin 150040, China; yuedaran@163.com

**Keywords:** cellulose nanofibrils, coir, ultrasonication, rheology

## Abstract

In this report, the isolation and rheological characterization of cellulose nanofibrils from coir (CNFs-1) were studied and compared with the CNFs from wood (CNFs-2) and cotton (CNFs-3). Cellulose nanofibrils were isolated successfully from coir fibers by chemical treatments followed by ultrasonic fibrillation. During ultrasonic processing, the size and the crystal structure of the CNFs were influenced by the raw materials. In comparison to CNFs-2 and CNFs-3, CNFs-1 from coir fibers presented diverse advantages, such as sufficient fibrillation with a low diameter distribution, in the range of 2–4 nm and high crystallinity. In the dynamic rheology study of CNFs-1, elastic behavior was observed and maintained due to the formation of gel-like steady network structures, which could not be easily deconstructured by frequency shearing and temperature changing. All results indicated that coir fibers could be used as a valuable resource for the preparation of CNFs, which exhibited comparable properties with those isolated from wood, in regard to morphology and rheological properties. This work provides a basis for further advanced applications using the CNFs isolated from coir fibers.

## 1. Introduction

Nanocellulose is considered the most promising bionanoparticle of the 21st century because of its sustainability, biocompatibility, biodegradability and capacity for broad chemical modification and assembly. Nanocelluloses, which include cellulose nanofibrils (CNFs) and nanocrystalline cellulose (NCC), can be obtained from lignocellulosic renewable resources, such as wood, cotton, and bamboo [1,2,3,4]. Compared with NCC, CNFs exhibit more attractive properties, such as a large specific surface area, high elastic modulus and high aspect ratio. In addition, their ability to act as a significant reinforcement at low filler loading levels stimulates their application as reinforcing agents in the polymer nanocomposite sector [5,6]. 

Owing to their unique morphology, mechanical properties, and negligible thermal expansion, CNFs have attracted significant interest in materials science [7]. Different methods have been applied for the preparation of CNFs, such as grinding, high-pressure homogenization, high-intensity ultrasonication and enzymatic methods [8,9,10]. High-intensity ultrasonication is a relatively effective method for disintegrating CNFs from cellulosic materials because it is convenient to operate and does not significantly affect the fiber properties of the material [11]. During ultrasonic treatment, the mechanical oscillating power, caused by cavitation, breaks down the interaction force between cellulose microfibrils and ultimately, disintegrates fibers into nanofibrils. Several reports on CNFs isolated from various lignocellulosic biomass by high-intensity ultrasonication have thus far been reported in the literature [12,13,14,15,16]. The fundamental properties of native CNFs, such as morphology, crystallinity, aspect ratio and surface chemistry vary depending on the source of raw materials and the extraction process [4,17,18]. These raw materials may alter the interaction between CNFs and influence the rheological behavior.

Coir is a renewable and biodegradable lignocellulosic fiber, extracted from the outer shell, or husk, of fruits of the coconut palm, which is an important and popular fruit tree, widely cultivated in tropical regions [19]. Coconut trees have been cultivated for more than 2000 years in the south of China. There is around 600 thousand acres of planting area in the Hainan province of China [20]. After harvesting of the fruits, a large amount of coir is normally generated as waste, which is abandoned for natural decay or disposed by burning. This has resulted in various environmental problems. Only a small part of coir fibers are used to prepare floor mats, mattresses, ropes, and composites [21,22,23,24,25]. Compared to other natural fibers, coir fibers have weather resistance, wettability, resistance to dampness, resiliency, toughness, and abrasion due to their low cellulose and hemi-cellulose content, and high lignin content [26]. To better utilize coir fibers, an evaluation of coir fibers as a non-wood cellulose resource for the production of nanocellulose deserves a systematic investigation. Several researchers have investigated its structure and properties, and several processes and products have been reported that utilize coir fibers as a raw material in the preparation of NCC [27,28]. However, studies on the preparation of CNFs from coir fiber have rarely been reported.

In the study of the relationship between structure and properties, the rheological properties of materials have been paid much attention, because they not only provide necessary material parameters for processing, but also provide information about the internal structure of materials [29]. Dynamic rheological measurements are usually carried out under small strain conditions and do not affect or destroy the microstructure of the material [30]. The viscous and elastic data of the system can be provided at the same time by the small amplitude oscillating flow field, which is closely related to the morphology characteristics of the dispersed phase [31,32,33]. A more detailed understanding of the rheological behavior of CNFs dispersions can aid in the development of nanocellulose composite materials with controlled properties. 

In this study, CNFs-1 was disintegrated from coir fibers by chemical purification combined with high-intensity ultrasonic treatment. Wood and cotton were also used as raw materials to prepare CNFs using the same method. The resulted CNFs are referred to as CNFs-2 and CNF-3, respectively. The morphology and crystal structure of all three obtained CNFs and their intermediate products were characterized and compared in detail by means of scanning electron microscopy (SEM), transmission electron microscopy (TEM), and X-ray diffraction. Moreover, to have a more comprehensive understanding of the CNFs gelation networks, the dynamic rheological properties of the CNFs were analyzed using a rheometer. 

## 2. Experimental

### 2.1. Raw Materials

Coir fibers, wood, and cotton were used as native cellulose fibers. Coir was collected from Hainan Province, China. Coir powder was sieved under 60 mesh, which is equivalent to a hole size of 0.25 mm. Similarly, wood powder from fast-growing poplar was sieved under 60 mesh. Long-staple cotton was collected from Urumqi, Xinjiang Province, China. After they were washed with distilled water, all samples were air-dried and then stored at room temperature. Benzene, ethanol (95%), sodium chlorite (NaClO_2_), acetic acid, KOH, and other chemicals were of laboratory grade and used without further purification.

### 2.2. Chemical Purification

To obtain the CNFs, the original aggregate structures of wood cell walls were disassembled, in order to remove extractives, lignin, and hemicelluloses by chemical treatment. Chemical purification was performed in accordance with previous studies [34,35]. First, 5 g of coir fibers were dewaxed in a Soxhlet apparatus using a 150 mL mixture of benzene and ethanol (95%) with a volume ratio of 2/1, for 6 h, for extractive removal. Second, samples were treated with an acidified NaClO_2_ solution (0.6 g NaClO_2_ dissolved in 65 mL distilled water and the pH of solution is within 4–5 treated with glacial acetic acid) at 75 °C, for 1 h, for lignin removal. The process was repeated 4 times until the samples turned white. Third, samples were treated in 100 mL (5 wt %) potassium hydroxide (KOH) solution at room temperature overnight and then treated at 90 °C for 2 h to leach the residual starch, pectin, and hemicelluloses. For thorough removal of lignin, hemicelluloses, residual starch, and pectin, the samples were further treated with NaClO_2_ at 75 °C for 1 h, and then treated with 5 wt % KOH at 90 °C for 2 h. After all chemical treatments, the samples were filtered and rinsed thoroughly with distilled water until the residues became neutral. The samples were maintained in a water-swollen state the entire time to prevent strong hydrogen bonds from being generated between the cellulose bundles after the chemical treatments [12]. The wood fibers and cotton fibers were also treated with the aforementioned process. The obtained purified cellulose fibrils from coir fibers, wood and cotton are referred to as PCFs-1, PCFs-2 and PCFs-3, respectively.

### 2.3. Ultrasonic Fibrillation

All three PCFs suspensions in water with a concentration at 0.8 wt % were placed in a high-speed agitator with high-speed shearing for a specific period. Then, the well dispersed PCFs suspensions were then sonicated for 20 min using an ultrasonic processor (SCIENTZ-1200E, Ningbo Scientz Biotechnology Co., Ltd., Ningbo, China) at 800 W to isolate the nanofibrils. The ultrasonic treatment was conducted in an ice bath; the distance from the tip of the ultrasonic generator probe to the beaker bottom was set to about 15 mm. Ice was maintained throughout the entire ultrasonication process. The resulted CNFs from PCFs-1, PCFs-2 and PCFs-3 are referred to as CNFs-1, CNFs-2 and CNFs-3, respectively. After chemical purification and ultrasonic fibrillation, yields of CNFs-1, CNFs-2 and CNFs-3 were 29%, 32% and 69%, respectively.

### 2.4. Microscopy Observation

Suspensions of the PCFs and CNFs were subjected to freeze-drying. The obtained aerogels were coated with gold and then SEM images were taking using a FEI XL-30 electron microscope (Thermo Fisher Scientific, Waltham, MA, USA). TEM images of the CNFs were taken using a FEI Tecnai F20 electron microscope (Thermo Fisher Scientific, Waltham, MA, USA) at 187 kV acceleration voltage. The diluted CNFs suspensions were dropped on electron microscopy grids with carbon coating and subsequently stained with a 1% phosphotungstic acid solution. Nano Measurer 1.2 (Department of Chemistry, Fudan Univ., Shanghai, China) was used to calculate the diameters of CNFs from the TEM images. The diameters were also illustrated based on the data of several CNFs and then used to analyze the diameter distributions.

### 2.5. Crystal Structure Analysis

The X-ray patterns of raw materials, PCFs and CNFs, were measured using a D/max 2200 X-ray diffractometer (Rigaku, Tokyo, Japan) with Ni-filtered Cu Kα radiation (λ = 1.5406 Å) at 40 kV and 30 mA. The X-ray radiation was collected from 2θ = 5° to 40° at a scanning rate of 5°/min. The crystallinity index (*CI*) was calculated using the Segal method. The equation is as follows:

$$CI\left(\%\right)=\frac{I_{200}-I_{am}}{I_{200}}\times 100$$

where $I_{200}$ (which represents both the crystalline and amorphous material) is the height of the 200 peak ($I_{200}$, 2θ = 22.6°) and $I_{\mathrm{am}}$ (which represents an amorphous material, 2θ = 18°) is the lowest height between the peaks at 200 and 110.

### 2.6. Dynamic Rheology Analysis

A stress-controlled AR-2000 rheometer (TA Instruments, Newcastle, PA, USA) was used to examine the rheological behavior of the CNFs suspensions. All measurements were conducted with an aluminum parallel-plate geometry (diameter, 40 mm; gap, 1 mm). Due to the significantly higher viscosity at concentrations higher than 1%, all the samples were measured at a concentration of 0.8%, which was the critical gelation concentration [36]. First, CNFs suspensions were subjected to strain sweeps to obtain the values of strain amplitude, which is used to ensure the dynamic oscillatory deformation within the linear viscoelastic region. The strain amplitude value selected was 0.01, which was in the linear viscoelastic region for all samples. CNFs suspensions were then subjected to frequency ramping and temperature ramping studies. Frequency sweeps were performed at 25 °C, from 0.1 to 100 rad/s. For temperature sweeps, measurements were conducted at 1 Hz, from 20 to 80 °C, at a heating rate of 2 °C/min. The edge of the sample was covered with a thin layer of paraffin oil to prevent water from evaporating during measurements.

## 3. Results and Discussion

### 3.1. Morphological Changes and Size Distribution of CNFs from Different Raw Materials

**Figure 1:**
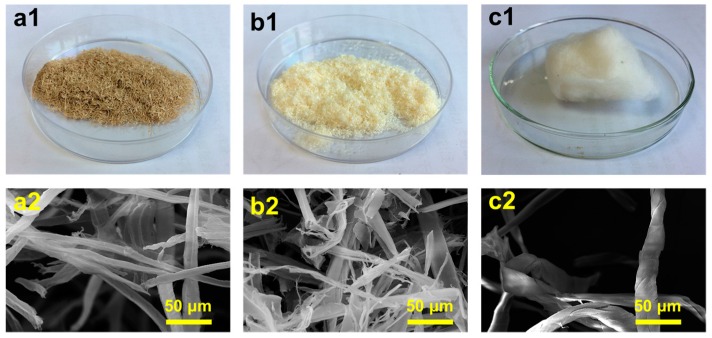
Digital photos showing (**a1**) coir, (**b1**) wood and (**c1**) cotton and SEM images of (**a2**) PCFs-1, (**b2**) PCFs-2 and (**c2**) PCFs-3.

Figure 1 presents a comparison of the digital photos of the three raw materials and SEM images of the purified cellulose fibrils (PCFs) from coir, wood, and cotton. The coir fibers show a brown colour due to the high content of lignin (Table S1 in Supplementary Materials). The individual fiber cells are narrow and hollow with thick walls made of cellulose (Figure S1 in Supplementary Materials). After repeated treatments with the acidified NaClO_2_ and KOH solution, all three PCFs showed a bar-like structure and smooth surfaces. This observation indicates that lignin and hemicellulose were almost completely removed. Furthermore, all three raw materials were separated into individual micro-sized fibers after the chemical treatments; these micro-sized cellulose fibers are composed of strong hydrogen bonding nanofibers, which could be disintegrated into individual nanofibers after ultrasonic treatments.

**Figure 2:**
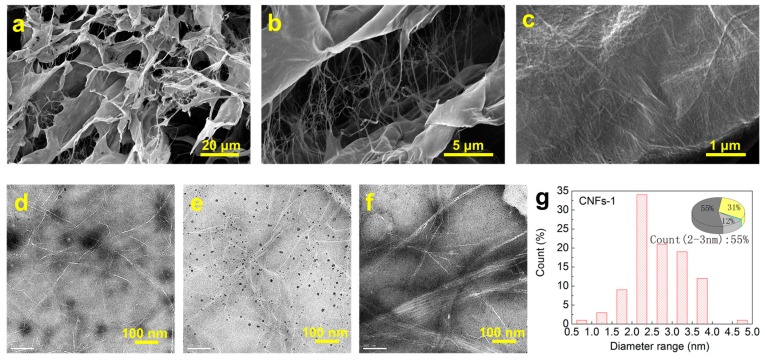
(**a**–**c**) SEM images of CNFs-1 under different magnifications; (**d**–**e**) TEM images of (**d**) CNFs-1; (**e**) CNFs-2 and (**f**) CNFs-3; (**g**) diameter distribution of CNFs-1 counted from the TEM images.

After freeze-drying from CNFs-1, the obtained aerogel exhibited a network structure with pores consisting of a large number of single nanofibrils, fiber clusters, and lamellar structures (Figure 2a). Figure 2b clearly shows a large number of single nanofibrils between the fiber cluster and the lamellar structure. These single nanofibrils connect the fiber cluster and the lamellar structure. A strong connection is formed between the two, which can efficiently improve the structural strength of the aerogel. As shown in Figure 2c, the lamellar structure with high interlacing density is formed by the intertwining of a large number of CNFs, and an interspace exists between the CNFs. The strong hydrogen caused self-assembly of the nanofibrils into clusters and lamellar structures during freeze-drying. The aerogels dried from CNFs-2 and CNFs-3 showed similar porous structures (Figure S2 in Supplementary Materials). Figure 2d–f show a comparison between the TEM images of the isolated nanofibers after ultrasonication of the three kinds of PCFs. The nanofibers of CNFs-1 and CNFs-2 were well-individualized and could easily be observed. This indicates that the microfibrils from coir and wood were separated completely, and no unseparated microfibrils were present. However, the sample of CNFs-3 showed the presence of fiber clusters and microfibrils, indicating insufficient isolation of the nanofibrils from cotton. Cotton fiber had a high cellulose content and high crystallinity, hence the stronger hydrogen bonding between cotton cellulose fibrils compared to other raw materials [37]. Consequently, the cotton CNFs could not achieve a size and structure similar to those of coir and wood CNFs by ultrasonic treatment under similar conditions. In addition, the CNFs-3 prepared by defibrillation were likely to self-assemble and form a fibrous cluster as a result of the relatively stronger hydrogen bonding. Figure 2g presents a histogram of the diameter range distribution of CNFs-1. The figure shows that the diameter range of CNFs-1 is mainly concentrated within 2–3 nm and that the proportion is about 55%. CNFs-1 with a diameter range of 3–4 nm comprised 31% of the total, and those with a diameter range of 1–2 nm comprised 12% of the total. However, CNFs-1 with a diameter range of 4 nm and above, and a diameter range of 1 nm and below, comprised 1% of the total. Thus, the majority (86%) of CNFs-1 have diameters in the range of 2–4 nm. Almost no coarse fibers were observed. This finding indicates that the diameter range distribution of CNFs-1 prepared by chemical pretreatment with ultrasonic treatment is mainly in the rage of 2-4 nm. These values agree rather well with previous reports [38]. In contrast, the nanofibrils from CNFs-2 and CNFs-3 have a relatively large size distribution and large average diameter (Figure S3 in Supplementary Materials). In particular, a certain portion of nanofibrils from CNFs-3 have diameters of a few dozens of nm.

### 3.2. Crystal Structure of CNFs from Different Raw Materials

**Figure 3:**
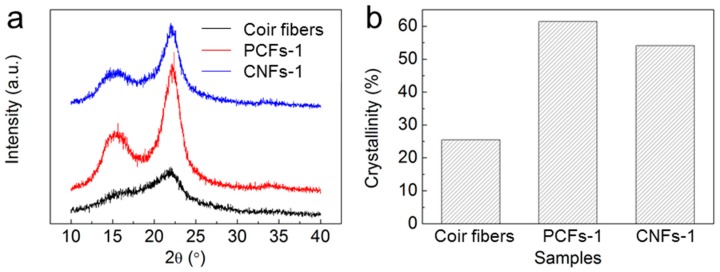
X-ray diffraction patterns (**a**) and crystallinities (**b**) of original untreated coir fibers, PCF-1 and CNF-1.

X-ray diffraction studies of the treated and untreated cellulose fibers were performed to investigate the crystalline behavior of the CNFs (Figure 3 and Figure S4 in Supplementary Materials). All samples had a lower peak at 2θ = 16.5 and a narrow peak at 2θ = 22.6. These results show the typical crystalline I form [39] and indicate that the native cellulose crystal integrity remained unchanged during chemical purification and ultrasonic fibrillation. The relative crystallinity was calculated from the X-ray diffraction patterns. The apparent crystallinity of the original untreated coir fibers was 26%. After chemical purification and high-speed shearing, the apparent crystallinity of PCFs-1 from coir fibers increased to 61%, owing to the removal of most of the extractives, lignin, and hemicelluloses. After the ultrasonic defibrillation, a slight decrease was found for CNFs-1 compared with the PCFs of coir fibers. After ultrasonic treatment, the crystallinity of CNFs-1 was fairly close to that of PCFs, with a value of 54%, implying that the ultrasonic treatment caused little damage to the crystalline region of the nanofibers. In contrast, crystallinities of CNFs-2 and CNFs-3 decreased significantly after ultrasonic treatment, indicating severe damage to the crystalline region (Figure S3 in Supplementary Materials). Thus, in comparison to wood and cotton, coir fibers show some advantages for the preparation of CNFs via the applied ultrasonic fibrillation methodology. Moreover, the high crystallinity of CNFs-1 will provide a high Young’s modulus, which could make CNFs-1 more effective in providing reinforcement for composites [40].

### 3.3. Dynamic Rheology of CNFs from Different Raw Materials

**Figure 4:**
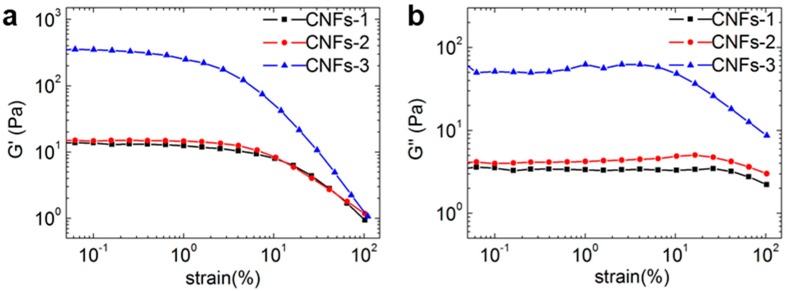
Strain ramping study for all three CNFs suspensions. (**a**) storage modulus (*G′*) and (**b**) loss modulus (*G″*).

Generally, dynamic strain sweep is required to determine the dependence of the strain modulus on the moduli of samples for the determination of the linear viscoelastic region. As shown in Figure 4, when the strain amplitude value was less than 1%, the storage modulus (*G′*) and loss modulus (*G″*) curves of three CNFs suspensions were basically maintained. *G′* and *G″* exhibited no influence on strain amplitude. With an increase in strain amplitude, the *G′* and *G″* of all three CNFs suspensions decreased gradually, and the suspensions exhibited nonlinear behavior. Therefore, the strain amplitude selected was 0.01 for the subsequent measurements of frequency sweep and temperature sweep. This selection was in the linear viscoelastic region for all samples. In the linear viscoelastic region, *G′* was higher than *G″*, which indicates a solid- or gel-like elastic behaviour for all three CNFs. At a certain strain amplitude, both the moduli started to decrease due to a breakdown or disruption of the ‘‘elastic’’ network formed by the nanofibrils. In comparison to CNFs-1 and CNFs-2, CNFs-3 had the highest *G′* and *G″* within the linear viscoelastic region. This is assumed to be related to the longer nanofibers isolated from cotton which create a more stable entangled network during shearing. Due to the similar morphology and surface chemistry, CNFs-1 exhibited closer strain amplitude-dependent rheological behavior to that of CNFs-2.

**Figure 5:**
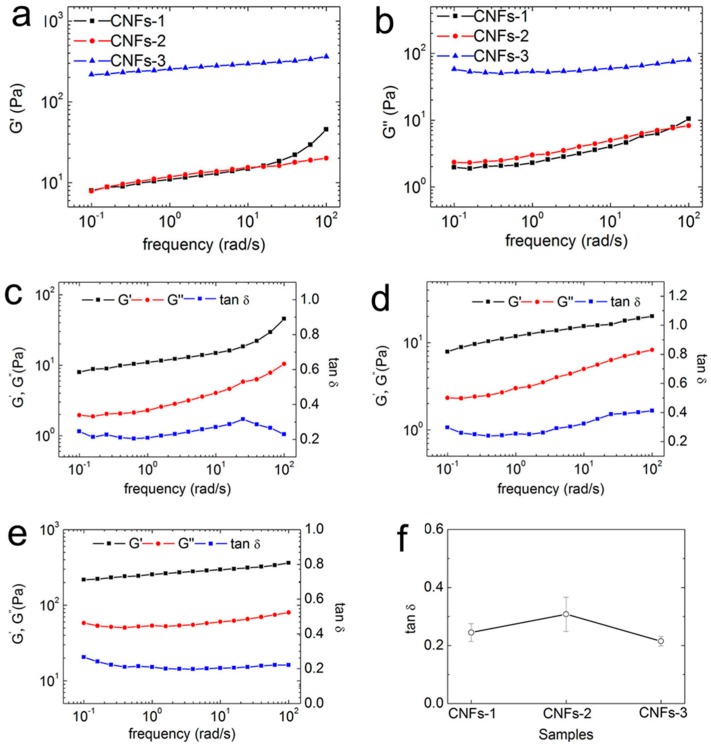
(**a**) Storage moduli and (**b**) loss moduli as functions of frequency, from 0.01 to 100 rad/s, for all three CNFs suspensions, at 25 °C. (**c**–**e**) Angular frequency dependence of the storage moduli, loss moduli, and loss tangents of (**c**) CNFs-1, (**d**) CNFs-2, and (**e**) CNFs-3 at 25 °C. (**f**) Average value of the loss tangents for all three CNFs suspensions during the frequency ramping study in the frequency range of 0.01~100 rad/s, at 25 °C.

The variations in the storage modulus (*G′*), loss modulus (*G″*), and tan δ = *G″*/*G′* (a dimensionless parameter that measures the ratio of loss modulus to storage modulus) with frequency were investigated (Figure 5). As shown in Figure 5a, the storage modulus of CNFs-3 varied from 10^2^ to 10^3^ Pa. The storage moduli of CNFs-1 and CNFs-2 varied from 10^1^ to 10^2^ Pa. The CNFs-3 suspension had much higher *G′* values than CNFs-1 and CNFs-2, mainly due to the formation of a more entangled CNFs network, because CNFs-3 had a larger aspect ratio. Furthermore, the TEM images of CNFs reveal that nanofibers, fibril aggregates, and microfibrils were present in CNFs-3 (Figure 2f). This occurrence also led to a higher storage modulus for the CNFs-3 suspension, in comparison to CNFs-1 and CNFs-2. During the entire frequency sweeps, the storage moduli of the CNFs-2 suspension and the CNFs-3 suspension increased slowly with an increase in frequency, and the increase in rate was minimal. This behavior illustrates that the CNFs-2 and CNFs-3 suspensions exhibited a certain degree of stability. CNFs are macromolecule materials and more hydroxyl groups are exposed to tight hydrogen bonding between fibrils during ultrasonic treatment. In addition, strong mechanical forces are produced by the intertwined CNFs. Thus, the CNFs suspension constituted a stable network structure. Hydrogen bonding between fibers and the specific surface prompted CNFs to reorganize rapidly when the network structure was damaged at a high frequency. So, the moduli of CNFs suspensions were affected slightly by the change in angular frequency. As the frequency increased, filament winding became closer and more orderly, which led to the slow rise in the storage moduli of the CNFs suspensions. However, the CNFs-1 suspension was less affected by the change in frequency within the low-angle range. The storage modulus of the CNFs-1 suspension changed significantly at a high angular frequency. The slope of curve of the storage modulus of the CNFs-1 suspension increased obviously, and the storage modulus increased from ~15 to 40 Pa. The network structure had no time to reorganize quickly simultaneously because it was destroyed when the CNFs suspension rotated at a high frequency, as determined using a rheometer. Therefore, the storage modulus of the CNFs-1 suspension was considerably influenced by the increase in frequency. Figure 5b presents a diagram of the relationship between the loss modulus of the CNFs suspensions and the angular frequency. The loss moduli of the three CNFs suspensions showed similar changes in trend to the storage moduli. The strong hydrogen bonding between the CNFs and the strong mechanical forces caused by the intertwining of CNFs allowed the CNFs suspensions to constitute a stable network structure. Thus, the curve depicting the loss modulus exhibits a gradual increase.

Figure 5c–e show the angular frequency-dependent storage moduli, loss moduli, and tan δ of the three CNFs suspensions. Within the whole applied range of the angular frequency, the *G′* was always larger than *G″*, and no crossover between them was found, indicating an apparent elastic behavior. The graph shows that the storage moduli and the loss moduli of all CNFs suspensions increase with an increase in frequency. However, the increasing trend in the storage moduli and the loss moduli of CNFs-1 and CNFs-2 was more obvious than that of CNFs-3. Owing to the existence of nanofibers, fibril aggregates, and microfibrils, which were not separated completely in the CNFs-3 suspension, the components interacted with each other to form a more steady network structure in the CNFs-3 suspension, compared to CNFs-1 and CNFs-2. Consequently, the increase in angular frequency was not strong enough to destroy the network structure. The tan δ values of the CNFs-1 and CNFs-2 suspensions ranged from 0.2 to 0.4. The tan δ values of the CNFs-3 suspension ranged from 0.2 to 0.28. Moreover, the tan δ values of CNFs-1 were less than 0.4 throughout the entire frequency ramping study, suggesting that the CNFs-1 suspension had already been in a gel form. The plot of the average value of tan δ is presented in Figure 5f, whereas tan δ is a dimensionless parameter that measures the ratio of the loss modulus to the storage modulus. The average values and standard deviations of tan δ for the suspensions of CNFs-1, CNFs-2 and CNFs-3 were 0.2 and 0.027, 0.27 and 0.026, and 0.19 and 0.015, respectively. These values indicate that the suspension was predominantly in an elastic state. The aforementioned results show that a steady network structure and an elastic gel were formed for the three CNFs suspensions.

**Figure 6:**
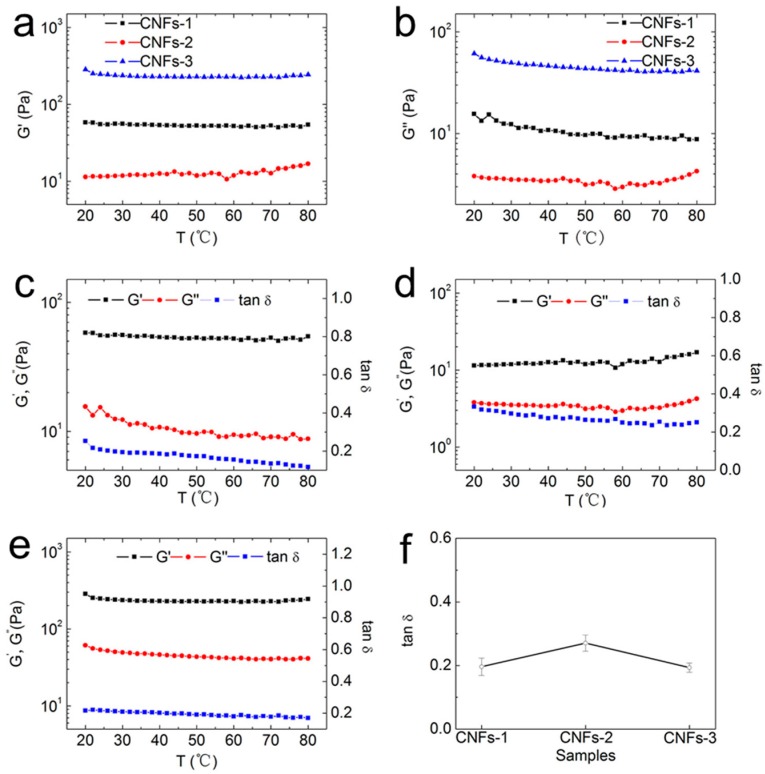
(**a**) Storage moduli and (**b**) loss moduli as functions of temperature, ranging from 20 to 80 °C, at a heating rate of 2 °C/min, for all three CNFs suspensions, at 1 Hz. (**c**–**e**) Temperature-dependent storage moduli, loss moduli, and loss tangents of (**c**) CNFs-1; (**d**) CNFs-2, and (**e**) CNFs-3, at 1 Hz, with a heating rate of 2 °C/min. (**f**) Average values of the loss tangent during the frequency ramping study in the temperature range of 20~80 °C.

The variations in *G′* and *G″* with temperature were also investigated (Figure 6). As shown in Figure 6a, all three CNFs suspensions remained flat with an increase in temperature. The ultrasonic disintegrated CNFs in this study showed inactive surface characteristics, larger aspect ratios, and high flexibility. Thus, the chemical interactions between CNFs, and the hydrogen bonds between CNFs and water molecules, were relatively weak, whereas the physical entanglements between CNFs were quite strong. These physical entanglements were less dependent on the temperature. This is the reason for the observed phenomenon that all three CNFs suspensions at any temperature level applied in this research remained stable and displayed viscoelastic properties. The loss moduli of all three CNFs exhibited a change in trend similar to those of the storage moduli (Figure 6b). Figure 6c–e show that increases in the storage moduli and loss moduli of different CNFs were not obvious during heating. This result indicates that thermal motion was always weaker than hydrogen bonding and mechanical resistance caused by cross-links among nanofibrils [36]. Throughout the entire heating experiment, the storage modulus was always higher than the loss modulus, and the tan delta values of the CNFs suspensions prepared from the three raw materials were less than 0.3. All data showed that the CNFs suspensions clearly exhibited elastic behavior. Simultaneously, the storage moduli and loss moduli of the three CNFs suspensions remained constant. This finding suggests that a high intensity network structure, which could not be influenced easily by temperature, was formed in the CNFs suspensions. The plot of the average values of tan δ in the temperature ramping study is presented in Figure 6f. The average values and standard deviations of tan δ for suspensions of CNFs-1, CNFs-2 and CNFs-3 were 0.2 and 0.027, 0.27 and 0.026, and 0.19 and 0.015, respectively. The values indicate that the suspension had a predominantly elastic behavior. The aforementioned results suggest that a steady network structure and strong gels were formed. The strong network structure and elastic behavior could not be easily influenced by an increase in temperature.

## 4. Conclusions

In summary, an efficient method for the preparation of CNFs-1 from a kind of agricultural residue, coir fibers, showing a narrow diameter range of 2–4 nm and relatively high crystallinity of 54%, was reported. CNFs-1 was obtained after a chemical purification process followed by ultrasonic fibrillation treatment in water. CNFs-2 was isolated from wood via the same methodology and exhibited similar morphology to that of CNFs-1. In contrast, the applied ultrasonic fibrillation treatment was not capable of disintegrating cotton into individual nanofibrils. The CNFs-3 from cotton contain a certain amount of fiber clusters and microfibrils, leading to a wide size distribution. This is due to the high cellulose content and strong hydrogen bonding of cotton. The reasonably long length of the starting cotton fibers is probably another reason. XRD measurements of all three CNFs exhibited a lower peak at 2θ = 16.5 and a narrow peak at 2θ = 22.6, which is characteristic for typical crystalline cellulose I. The viscoelasticity of all three CNFs suspensions were evaluated via rheometry to measure the storage (*G′*) and loss (*G″*) moduli. These three CNFs suspensions exhibited solid- or gel-like elastic behaviour, with *G′* being much higher than *G″* at all shear frequencies. In addition, all tan delta values of the three CNFs suspensions were less than 0.5, which also indicated that all three CNFs suspensions had predominantly elastic behavior. Furthermore, in comparsion to CNFs-1 and CNFs-2, the significant difference in the morphology of CNFs-3 from cotton led to relatively high *G′* and *G″* values, mainly due to the formation of a more entangled CNFs network. Moreover, CNFs from three raw materials showed excellent temperature-dependent rheological stability according to the result showing that the *G′* and *G″* remained constant with a temperature increase from 20 to 80 °C. To sum up, CNFs-1 from coir exhibited comparable properties with that isolated from wood, and showed various advantages compared with CNFs-3 from cotton. Thus, this provides a new approach for the high value-added utilization of coir fibers.

## Supplementary Materials

The Supplementary Materials are available online at http://www.mdpi.com/2073-4360/10/3/320/s1.
