# Supplementary material for: Isolation and Rheological Characterization of Cellulose Nanofibrils (CNFs) from Coir Fibers in Comparison to Wood and Cotton
Source: Polymers (Basel). 2018 Mar 14;10(3):320. doi: 10.3390/polym10030320 (PMC6415033; doi:10.3390/polym10030320)
Supplement: Supplementary file 1 [file polymers-10-00320-s001.docx]

**Supporting Information**

Isolation and Rheological Characterization of Cellulose Nanofibrils (CNFs) from Coir fibers in Comparison to Wood and Cotton

Daran Yue·Xueren Qian*

(Key Laboratory of Bio-Based Materials Science and Technology of Ministry of Education, Northeast Forestry University, No. 26 Hexing Road, Xiangfang District, 150040, Harbin, China)

Chemical compositions measurement

The chemical compositions of the untreated coir fibers was measured in accordance with the standards of the Technical Association of Pulp and Paper Industry. And the wood fibers and cotton fibers were treated by the same method.

**Table S1** Chemical compositions of coir fibers, wood fibers and cotton fibers.

| Samples | α-Cellulose(%) | Hemicelluloses(%)_ | Acid soluble lignin(%) |
| --- | --- | --- | --- |
| Coir fibers | 39.9 | 20.9 | 30.5 |
| Wood fibers | 47.5 | 21.1 | 11.7 |
| Cotton fibers | 79.8 | 2.3 | 0.5 |

The chemical compositions of raw materials are presented in Table S1. Coir fibers have highest content of lignin compared to wood fibers and cotton fibers. This is constent with some other reports ([D. Verma & Gope, 2015](#_ENREF_1); [Deepak Verma, Gope, Maheshwari, Shandilya, & Gupta, 2013](#_ENREF_2)).


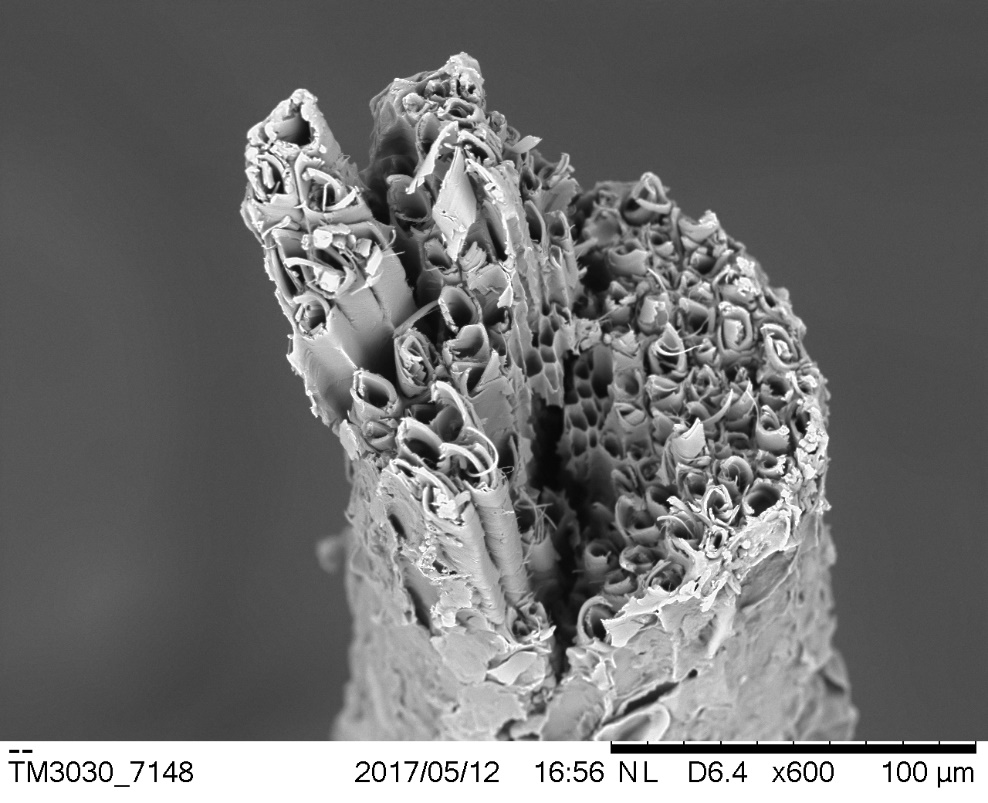


**Fig. S1** The SEM image of fractured surface of coir fiber


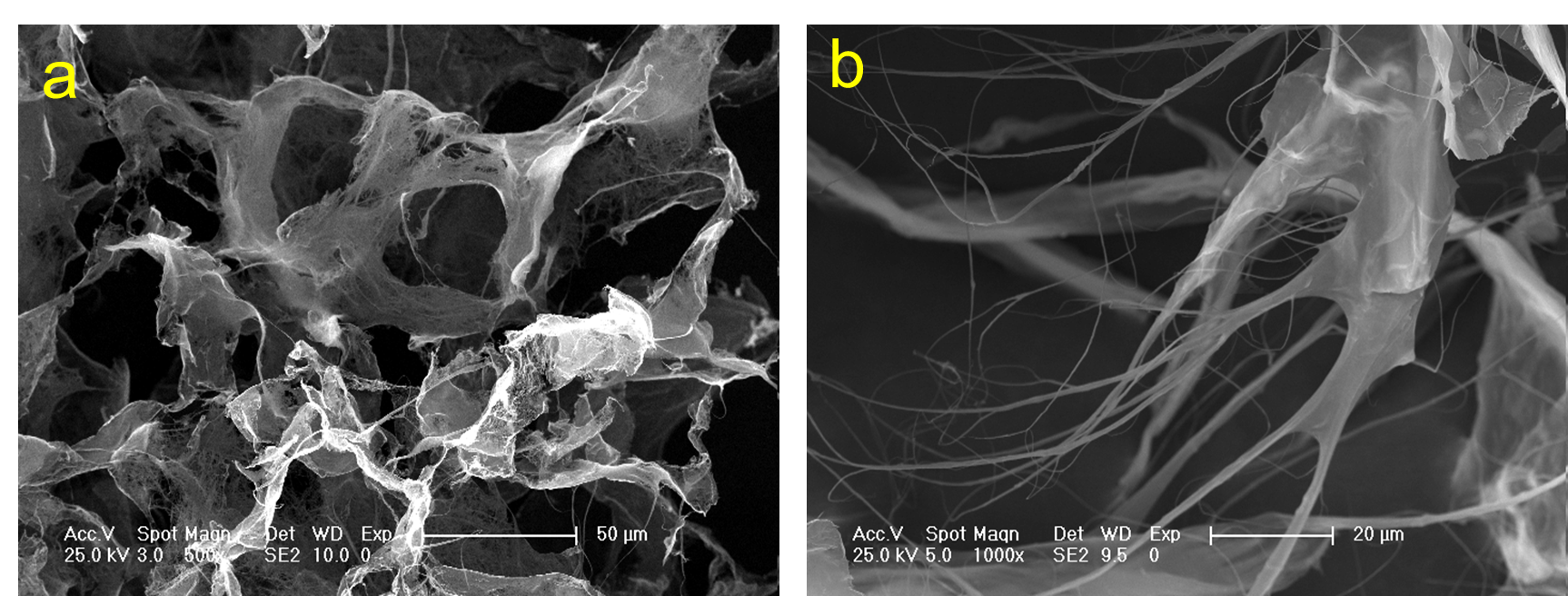


**Fig. S2** SEM images of (a) CNFs-2 and (b)CNFs-3.


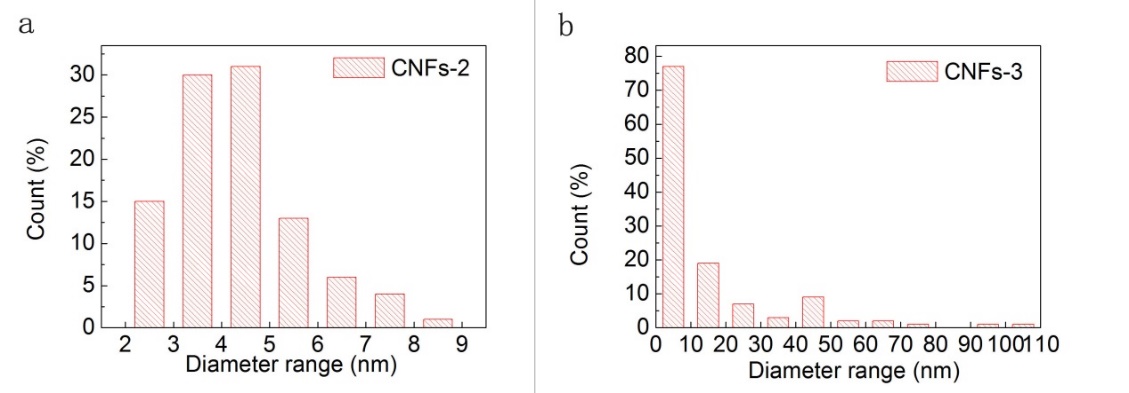


**Fig. S3** Diameter range of CNFs-2 and CNFs-3 counted from the TEM images


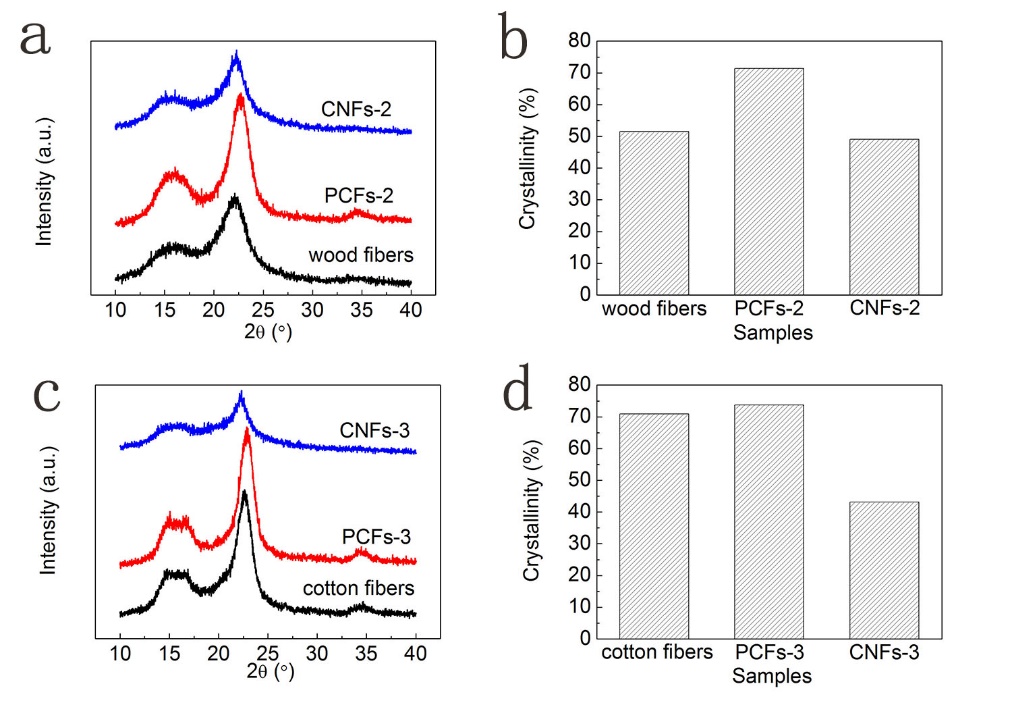


**Fig. S4** (a-b) X-ray diffraction patterns (a) and crystallinities (b) of original untreated wood fibers, PCF-2 and CNF‑2. (c-d) X-ray diffraction patterns (a) and crystallinities (b) of original untreated cotton fibers, PCF-3 and CNF‑3.

**References**

Verma, D., & Gope, P. C. (2015). *The use of coir/coconut fibers as reinforcements in composites*.

Verma, Deepak, Gope, P. C., Maheshwari, M. K., Shandilya, A., & Gupta, A. (2013). Coir Fiber Reinforcement and Application in Polymer Composites: A Review. *Journal of Hubei University of Automotive Technology, 4*(2), 263-276.
